# LLM-Powered Evolutionary System for Generating Large-Scale Databases in Speech-Related Psychiatric Conditions

**DOI:** 10.1192/j.eurpsy.2025.589

**Published:** 2025-08-26

**Authors:** E. Gutierrez Alvarez, P. Cano, J. M. Vera, E. DeFraites

**Affiliations:** 1 Universidad Politécnica de Madrid, Madrid, Spain; 2 MIT linQ - Massachusetts Institute of Technology, Cambridge; 3 Mental Health Intensive Case Management, Greater Los Angeles VA Healthcare System; 4 Department of Psychiatry, UCLA - University of California, Los Angeles, Los Angeles; 5 MIT linQ - Massachusetts Institute of Technology, Cambridge, United States

## Abstract

**Introduction:**

In clinical studies on psychosis prediction, small sample sizes have been a persistent issue. Most studies rely on limited data, lack cross-validation, and use poor model strategies, leading to overfitting and overestimated accuracy. This challenge also affects traditional studies, where recruiting few participants introduces biases. Data harmonization is another hurdle, especially in speech analysis, which is crucial in psychiatry for conditions like psychosis, aphasia, and PTSD, but suffers from inconsistent methodologies across databases.

**Objectives:**

Our goal was to develop an method using Large Language Models (LLMs) to create diverse, synthetic speech datasets, addressing these challenges: 1. Develop an evolutionary system for optimizing high-quality speech data generation. 2. Incorporate contrastive learning for improved model decision boundaries. 3. Provide a methodology for training classification models and conducting cross-cultural studies. 4. Create a large-scale, diverse database of synthetic psychiatric speech samples.

**Methods:**

**Results**

We presented a case study focused on the phenomenon of “Illogical Thinking,” a language disorder proven to correlate with psychosis risk. Results:

1. Top-performing LLMs: Claude Sonnet 3.5 and GPT-4.
2. Optimal prompt structure determined
3. Database size: 3,000 samples
4. Computational efficiency: 200 evolutionary steps, 400 API calls
5. High data quality and diversity
6. Useful rationales for developing explainable models

**Image 1:**

**Image 2:**

**Conclusions:**

Our findings suggest that this approach could significantly benefit psychiatric research by addressing the challenges of small sample sizes and data inconsistency. The method shows promise for creating more reliable and generalizable predictive models, which could lead to advancements in mental health care practices. The system’s flexibility indicates potential applications beyond our case study, possibly extending to other areas where data scarcity has impeded progress.

**Disclosure of Interest:**

None Declared

